# Supplementary material for: Content-rich biological network constructed by mining PubMed abstracts
Source: BMC Bioinformatics. 2004 Oct 8;5:147. doi: 10.1186/1471-2105-5-147 (PMC528731; doi:10.1186/1471-2105-5-147)
Supplement: Additional File 2 — The original results of the above study (non-essential files are deleted to keep the file size under the limit set by BMC bioinformatics). [file 1471-2105-5-147-S2.bz2 › chilibotAdditionalFile2/dip30/297ID7486683E1288/html/PIK3R1_IRS1.html]

 


 **PIK3R1** and **IRS1** 
  
Found 143 abstracts in PubMed, retrieved 30.  
 

 What does Google say? 
 PDF only 
| .edu only 

---

**Interactive relationship** (e.g. stimulation, inhibition, etc)

**Inhibitory relationship**- Our data show that the PI3K  [ **PIK3R1** ]  activity associated with IRS 1  [ **IRS1** ] , the docking of IRS 1  [ **IRS1** ]  to InR beta and the docking of p85 alpha  [ **PIK3R1** ]  to IRS 1  [ **IRS1** ]  are attenuated in the aged Snell dwarf.  Ref: 12020946 Mech Ageing Dev, 2002
- PDK1, mTOR, and MAPK inhibitors did not block insulin induced reduction of IRS 1  [ **IRS1** ] , suggesting that the PI3K  [ **PIK3R1** ]  serine kinase activity causes IRS 1  [ **IRS1** ]  serine phosphorylation and its commitment to proteasomal degradation.  Ref: 12594228 J Biol Chem, 2003
- Conversely, adenoviral driven expression of constitutively active PI3K  [ **PIK3R1** ]  induced an insulin independent reduction in IRS 1  [ **IRS1** ]  2 protein levels.  Ref: 12594228 J Biol Chem, 2003
- Desensitization was correlated to a reduction in insulin receptor substrate IRS 1  [ **IRS1** ]  and IRS  [ **IRS1** ]  2 protein levels, which was reversed by the PI3K  [ **PIK3R1** ]  inhibitor LY294002.  Ref: 12594228 J Biol Chem, 2003
- RESULTS Chronic treatment of insulin 100 nmol L induced a significant reduction in the phosphorylation and protein expression of IR ?,  **IRS1**  and IRS2, which therefore resulted in a downregulation of association of PI3K  [ **PIK3R1** ]  with IRS  [ **IRS1** ] .  Ref: 12511230 Acta Pharmacol Sin, 2003
- The MEK1 mutant, which activates ERK, markedly down regulated expression of the insulin receptor IR and its major substrates, IRS 1  [ **IRS1** ]  and IRS  [ **IRS1** ]  2, mRNA and protein, and in turn reduced tyrosine phosphorylation of IR as well as IRS 1  [ **IRS1** ]  and IRS  [ **IRS1** ]  2 and their associated phosphatidyl inositol 3 kinase PI3K  [ **PIK3R1** ]  activity.  Ref: 12554784 Mol Endocrinol, 2003
**Stimulatory relationship**- Co treatment of cells with insulin and LY294002, while reducing total IRS 1  [ **IRS1** ]  phosphorylation, increased its phosphotyrosine content, enhancing IRS 1  [ **IRS1** ]  PI3K  [ **PIK3R1** ]  association.  Ref: 12594228 J Biol Chem, 2003
- Exposure to IGF 1 for 30 minutes or E2 for 20 minutes increased insulin receptor substrate 1  [ **IRS1** ]  IRS 1  [ **IRS1** ]  association with the regulatory p85 subunit of PI3K  [ **PIK3R1** ] , enhanced tyrosine phosphorylation of p85, and increased PI3K  [ **PIK3R1** ]  activity.  Ref: 12701063 Metabolism, 2003
- Here, we report that insulin induces tyrosine phosphorylation of the insulin receptor and IRS 1  [ **IRS1** ]  and 2, increases binding of activated IRS 1  [ **IRS1** ]  and 2 to the regulatory subunit of PI3K  [ **PIK3R1** ] , and activates protein kinase B Akt, a downstream target of PI3K  [ **PIK3R1** ] .  Ref: 12540590 Diabetes, 2003
- In contrast, there was a high level of IRS  [ **IRS1** ]  2 expression and insulin stimulated tyrosyl phosphorylation as early as embryonic day 15 with robust PI3K  [ **PIK3R1** ]  binding and activation, which may enhance hepatocyte survival during the rapid growth phase of the liver.  Ref: 12891559 Gastroenterology, 2003
- IRS 1  [ **IRS1** ]  over expression in transgenic Tg mouse livers caused constitutive activation of Erk mitogen activated protein kinase MAPK and phosphatidylinositol 3 kinase PI3K  [ **PIK3R1** ]  resulting in significantly increased levels of DNA synthesis and larger hepatic masses relative to non transgenic non Tg littermates.  Ref: 12763374 J Hepatol, 2003
**Neutral relationship**- In contrast, metformin in pharmacological concentration 1 10 mmol L further inhibited tyrosine phosphorylation of IR ?,  **IRS1** , IRS2 and the interaction of PI3K  [ **PIK3R1** ]  with IRS  [ **IRS1** ] .  Ref: 12511230 Acta Pharmacol Sin, 2003
- Tyrosine phosphorylation of insulin receptor substrate 1  [ **IRS1** ]  is detected when enriched by immunoprecipitation with anti PI3K  [ **PIK3R1** ]  antibody.  Ref: 12577306 J Cell Biochem, 2003
- Contrarily, insulin induced IRS  [ **IRS1** ]  2 down regulation occurred via a PI3K  [ **PIK3R1** ]  mTOR pathway.  Ref: 12594228 J Biol Chem, 2003
- Using an immunohistochemical technique to detect PI, 5 triphosphate, the main product of PI3K  [ **PIK3R1** ]  activity, we further demonstrate that in the arcuate nucleus, insulin induced PI3K  [ **PIK3R1** ]  activity occurs preferentially within cells that contain IRS  [ **IRS1** ]  2.  Ref: 12540590 Diabetes, 2003

**Non-interactive relationship** (e.g. studied together, co-existance, homology, etc.)

- IRS 1  [ **IRS1** ]  content and insulin responsiveness of PI3K  [ **PIK3R1** ] , Akt, and S6K1 showed a transition to the adult phenotype during the first several postnatal weeks.  Ref: 12217903 Am J Physiol Endocrinol Metab, 2002
- IRS 1  [ **IRS1** ]  association with Grb2 and PI3K  [ **PIK3R1** ]  p85, which link IGFRI to the Ras MAPK and PI3K  [ **PIK3R1** ]  Akt pathways, respectively, was enhanced in both cell types in the presence of EN.  Ref: 12173038 Oncogene, 2002
- Insulin is a potent inducer of adipogenesis, and differentiation of adipocytes requires many components of the insulin signaling pathway, including the insulin receptor substrate IRS 1  [ **IRS1** ]  and phosphatidylinositol 3 kinase PI3K  [ **PIK3R1** ] .  Ref: 12807888 J Biol Chem< MedlineTA>J Biol Chem, 2002
- Importantly, weight loss in obese subjects normalizes PKClambda zeta activation and increases IRS 1  [ **IRS1** ]  phosphorylation and PI3K  [ **PIK3R1** ]  activity.  Ref: 12882908 DiabetesDiabetes, 2002
- The MKK6 mutant, which activates p38, moderately inhibited IRS 1  [ **IRS1** ]  and IRS  [ **IRS1** ]  2 expressions and IRS 1  [ **IRS1** ]  associated PI3K  [ **PIK3R1** ]  activity without exerting a significant effect on the IR.  Ref: 12554784 Mol Endocrinol, 2003
- METHODS We determined the expression and tyrosyl phosphorylation of the insulin receptor beta subunit IRbeta, IRS 1  [ **IRS1** ]  and IRS  [ **IRS1** ]  2, the binding of phosphatidylinositol 3 kinase PI3K  [ **PIK3R1** ] , and activation of the mitogen activated protein kinase MAPK pathway in the presence or absence of insulin stimulation in vivo during development and in the adult liver.  Ref: 12891559 Gastroenterology, 2003
- Shb overexpression resulted also in the assembly and activation of a multiunit complex consisting of at least Shb, IRS 1  [ **IRS1** ] , IRS  [ **IRS1** ]  2, FAK, and PI3K  [ **PIK3R1** ] .  Ref: 12520086 Mol Med, 2002
- Finally, the MKK7 mutant, which activates JNK, reduced tyrosine phosphorylation of IRS 1  [ **IRS1** ]  and IRS  [ **IRS1** ]  2 and IRS  [ **IRS1** ]  associated PI3K  [ **PIK3R1** ]  activity without affecting expression of the IR, IRS 1  [ **IRS1** ] , or IRS  [ **IRS1** ]  2.  Ref: 12554784 Mol Endocrinol, 2003
- Conclusion It is concluded that the Shb induced alterations in the IRS 1  [ **IRS1** ]  PI3K  [ **PIK3R1** ]  Akt pathway may be relevant to the understanding of growth and death patterns of insulin producing cells.  Ref: 12520086 Mol Med, 2002
- Insulin stimulated insulin receptor substrate IRS 1  [ **IRS1** ]  tyrosine phosphorylation and PI3K  [ **PIK3R1** ]  activity are impaired 40 50% in diabetic subjects compared with lean or obese subjects.  Ref: 12882908 DiabetesDiabetes, 2002
- Exposure to 100 nM IGF 1 for 10 min resulted in increased insulin receptor substrate 1  [ **IRS1** ]  associated PI3K  [ **PIK3R1** ]  activity and Akt kinase activity.  Ref: 12624428 Endocrine< MedlineTA>Endocrine, 2002
- docking of p 85 alpha to p110 alpha or p110 beta, and IRS  [ **IRS1** ]  2 associated PI3K  [ **PIK3R1** ]  activity.  Ref: 12020946 Mech Ageing Dev, 2002
- Insulin produced minimal activation of insulin receptor substrate IRS 1  [ **IRS1** ]  associated PI3K  [ **PIK3R1** ]  activity in fetal liver.  Ref: 12217903 Am J Physiol Endocrinol Metab, 2002
- Overexpression of a catalytically inactive dominant negative SHP 1 markedly attenuated the AT2 receptor mediated inhibition of IRS  [ **IRS1** ]  2 associated PI3K  [ **PIK3R1** ]  activity, Akt phosphorylation, and antiapoptotic effect induced by insulin.  Ref: 12198247 Mol Endocrinol, 2002
- AT2 receptor stimulation did not change insulin induced tyrosine phosphorylation of IRS  [ **IRS1** ]  2 or its association with the p85alpha subunit of PI3K  [ **PIK3R1** ] , but led to a significant reduction of insulin induced p85alpha phosphorylation.  Ref: 12198247 Mol Endocrinol, 2002
- In peripheral tissues, insulin signaling involves activation of the insulin receptor substrate IRS  [ **IRS1** ]  phosphatidylinositol 3 kinase PI3K  [ **PIK3R1** ]  enzyme system.  Ref: 12540590 Diabetes, 2003
- In the biopsies, insulin receptor kinase IRK activity, insulin receptor substrate IRS 1  [ **IRS1** ]  associated phosphatidylinositol 3 kinase PI3K  [ **PIK3R1** ]  activity, Ser 473 and Thr 308 phosphorylation of protein kinase B PKB, and protein expression of IRS 1  [ **IRS1** ] , IRS  [ **IRS1** ]  2, phosphoinositol dependent kinase 1 PDK 1, PKB, and GLUT 4 were determined.  Ref: 12196460 Diabetes, 2002
- Immunoprecipitation Western blot studies revealed that CLA inhibited IGF I induced phosphorylation of IGF IR and insulin receptor substrate IRS 1  [ **IRS1** ] , recruitment of the p85 regulatory subunit of phosphatidylinositol 3 kinase PI3K  [ **PIK3R1** ]  to IGF IR, IGF IR associated PI3K  [ **PIK3R1** ]  activity, and phosphorylated Akt and extracellular signal regulated kinase ERK 1 2 levels.  Ref: 12888657 J NutrJ Nutr, 2002
- docking of p85 alpha  [ **PIK3R1** ]  to IRS  [ **IRS1** ]  2.  Ref: 12020946 Mech Ageing Dev, 2002
- These results suggest that ceramide may induce insulin resistance via the suppression of IRS 1  [ **IRS1** ]  PI3K  [ **PIK3R1** ]  signaling, and subsequent activation of PKCzeta.  Ref: 12524657 Metabolism, 2003
- To test the hypothesis that hypothalamic insulin action involves intracellular PI3K  [ **PIK3R1** ]  signaling, we used histochemical and biochemical methods to determine the effect of insulin on hypothalamic IRS  [ **IRS1** ]  PI3K  [ **PIK3R1** ]  activity.  Ref: 12540590 Diabetes, 2003
- We investigated the effect of ceramide on insulin signaling pathways, such as insulin receptor IR beta subunit, insulin receptor substrate 1  [ **IRS1** ]  IRS 1  [ **IRS1** ] , phosphatidylinositol 3 kinase PI3K  [ **PIK3R1** ] , and protein kinase Czeta PKCzeta in rat adipocytes.  Ref: 12524657 Metabolism, 2003
- In summary, i PI3K  [ **PIK3R1** ]  mediates insulin induced reduction of IRS 1  [ **IRS1** ]  by phosphorylating it while a PI3K  [ **PIK3R1** ]  mTOR pathway controls insulin induced reduction of IRS  [ **IRS1** ]  2, ii in L6 cells, IRS  [ **IRS1** ]  2 is the major adapter molecule linking the insulin receptor to activation of PKB and MAPK, iii the mechanism of IRS 1  [ **IRS1** ]  2 down regulation is different in L6 cells compared with 3T3 L1 adipocytes.  Ref: 12594228 J Biol Chem, 2003
- Taken together, these results indicate that AT2 receptor mediated activation of SHP 1 and the consequent inhibition IRS  [ **IRS1** ]  2 associated PI3K  [ **PIK3R1** ]  activity contributed at least partly to the inhibition of Akt phosphorylation, thereby inducing apoptosis.  Ref: 12198247 Mol Endocrinol, 2002
- These findings support the hypothesis that the IRS  [ **IRS1** ]  PI3K  [ **PIK3R1** ]  pathway is a mediator of insulin action in the arcuate nucleus and, combined with recent evidence that leptin activates PI3K  [ **PIK3R1** ]  signaling in the hypothalamus, provide a plausible mechanism for neuronal cross talk between insulin and leptin signaling.  Ref: 12540590 Diabetes, 2003
- In conclusion, the reduction in IRS  [ **IRS1** ]  proteins via different PI3K  [ **PIK3R1** ]  mediated mechanisms contributes to the development of an insulin resistant state in L6 myoblasts.  Ref: 12594228 J Biol Chem, 2003
- Here we report that SP A treatment leads to rapid tyrosine specific phosphorylation of several important proteins in lung epithelial cells including insulin receptor substrate 1  [ **IRS1** ]  IRS 1  [ **IRS1** ] , an upstream activator of PI3K  [ **PIK3R1** ] .  Ref: 12413892 Exp Cell Res, 2002
- RINm5F cells overexpressing wild type GTK also exhibited reduced activation of IRS  [ **IRS1** ]  2, PI3K  [ **PIK3R1** ]  and AKT.  Ref: 12520087 Mol Med, 2002
- We also examined the effect of various concentrations of C 6 ceramide pretreatment on insulin induced autophosphorylation of the IR beta subunit, tyrosine phosphorylation of IRS 1  [ **IRS1** ] , enzyme activity of PI3K  [ **PIK3R1** ] , and membrane associated PKCzeta immunoreactivity.  Ref: 12524657 Metabolism, 2003
- In this study we present data suggesting that the InR beta IRS 1  [ **IRS1** ]  PI3K  [ **PIK3R1** ]  pathway is attenuated in the Snell dwarf mouse liver.  Ref: 12020946 Mech Ageing Dev, 2002
- Leptin stimulation activates JAK STAT, IRS 1  [ **IRS1** ]  PI3K  [ **PIK3R1** ]  and MAPK signalling pathways.  Ref: 12823272 Clin Exp Immunol, 2003
- Insulin receptor substrate IRS 1  [ **IRS1** ]  was associated with PI3K  [ **PIK3R1** ]  activity, which is necessary for Akt activation by insulin, and was reduced in obese muscles, and this was accompanied by decreased IRS 1  [ **IRS1** ]  expression.  Ref: 12663464 Diabetes, 2003
- These include a decreased IRS  [ **IRS1** ]  2 pool level, a decrease in PI3K  [ **PIK3R1** ]  activity and its association with IRS  [ **IRS1** ]  2 and decreased docking of p85alpha to IRS  [ **IRS1** ]  2.  Ref: 12020945 Mech Ageing Dev, 2002
- Our data also suggest a preferential docking of IRS  [ **IRS1** ]  2 p85 alpha  [ **PIK3R1** ]  p110 alpha in the aged dwarf liver and IRS  [ **IRS1** ]  2 p85 alpha  [ **PIK3R1** ]  p110 beta in the aged control.  Ref: 12020945 Mech Ageing Dev, 2002
- However, increased IRS  [ **IRS1** ]  3 expression blocked glucose IGF 1 induced IRS  [ **IRS1** ]  2 translocation from the cytosol to the plasma membrane, dampening IRS  [ **IRS1** ]  2 IGF 1R interaction and subsequent activation of the PI3K  [ **PIK3R1** ]  PKB GSK3 signaling pathway.  Ref: 12850284 Mol Cell Endocrinol, 2003
- Therefore, we have characterized survival enhancing signaling activated by SP A leading from SPAR through IRS 1  [ **IRS1** ] , PI3K  [ **PIK3R1** ] , PKB Akt, and FKHR.  Ref: 12413892 Exp Cell Res, 2002
- ... IRS 1  [ **IRS1** ]  associated PI3K  [ **PIK3R1** ]  activity was not significantly influenced.  Ref: 12198247 Mol Endocrinol, 2002
- IRS  [ **IRS1** ]  2 and phosphotyrosine associated PI3K  [ **PIK3R1** ]  activity were increased 3.5 and 4.8 fold, respectively, by insulin in glycerol infused rats but only 1.6 and 2.3 fold in lipid infused rats.  Ref: 12095990 J Biol Chem, 2002
- These data emphasize the importance of IRS  [ **IRS1** ]  2 PI3K  [ **PIK3R1** ]  PKB signal transduction for beta cell growth and survival.  Ref: 12850284 Mol Cell Endocrinol, 2003
- We then identified an increase in JAK2,  **IRS1**  and IRS2 tyrosine phosphorylation and in their association with p85, a subunit of PI3K  [ **PIK3R1** ] .  Ref: 12706299 Mol Cell Endocrinol, 2003
- This was accompanied by reduced expression of IRS  [ **IRS1** ]  2 and attenuated insulin stimulated recruitment of PI3K  [ **PIK3R1** ]  to IRS 1  [ **IRS1** ]  and IRS  [ **IRS1** ]  2, yet insulin stimulated PKB activity and phosphorylation of eNOS at Ser1177 were unaffected.  Ref: 12644458 J Biol Chem, 2003
- Insulin stimulated IRS 1  [ **IRS1** ]  associated PI3K  [ **PIK3R1** ]  activity in muscle of glycerol infused rats 2.4 fold but had no effect in lipid infused rats.  Ref: 12095990 J Biol Chem, 2002
- At the molecular level, impaired activation of the IR IRS  [ **IRS1** ]  PI3K  [ **PIK3R1** ]  pathway and a significant decrease both in the levels and in insulin stimulated activation of the serine threonine kinase Akt were observed.  Ref: 12767044 J Cell Physiol, 2003
- Pretreatment with C 6 ceramide significantly reduced autophosphorylation of the IR beta subunit, tyrosine phosphorylation of IRS 1  [ **IRS1** ] , and enzyme activity of PI3K  [ **PIK3R1** ] .  Ref: 12524657 Metabolism, 2003
